# Clinical analysis and pluripotent stem cells-based model reveal possible impacts of ACE2 and lung progenitor cells on infants vulnerable to COVID-19

**DOI:** 10.7150/thno.53136

**Published:** 2021-01-01

**Authors:** Zhao Zhang, Liyan Guo, Xiaoxia Lu, Che Zhang, Li Huang, Xianfeng Wang, Fuyu Duan, Huiying Liang, Peikai Chen, Liang Zeng, Jianbo Shao, Hui Li, Le Li, Li Liu, Cheng Li, Jinqiu Zhang, Chui Yan Ma, Ka Yi Kwan, Wei Liu, Yi Xu, Xiaoqiong Gu, Hua Jiang, Hui Du, Ting Zhang, Yanheng Wu, Guangyin Yu, Junhui Chen, Ruibang Luo, Can Liao, Hung-fat Tse, Zhiwei Chen, Huanhuan Joyce Chen, Huimin Xia, Qizhou Lian

**Affiliations:** 1 Prenatal Diagnostic Center and Cord Blood Bank, Guangzhou Women and Children's Medical Center, Guangzhou Medical University, Guangzhou, China; 2 Guangdong Provincial Children's Medical Research Center, Guangzhou Women and Children's Medical Center, Guangzhou Medical University, Guangzhou, China; 3 Department of Pathology, Guangzhou Women and Children's Medical Center, Guangzhou Medical University, Guangzhou, China; 4 Department of Clinical Biological Resource Bank, Guangzhou Women and Children's Medical Center, Guangzhou Medical University, Guangzhou, China; 5 Department of Surgery, Guangzhou Women and Children's Medical Center, Guangzhou Medical University, Guangzhou, China; 6 Department of Haematology, Guangzhou Women and Children's Medical Center, Guangzhou Medical University, Guangzhou, China; 7 Department of Emergency Medicine, Guangzhou Women and Children's Medical Center, Guangzhou Medical University, Guangzhou, China; 8 Department of Medicine, the University of Hong Kong, Hong Kong SAR, China; 9 AIDS Institute and Department of Microbiology, Li Ka Shing Faculty of Medicine, the University of Hong Kong, Hong Kong SAR, China; 10 School of Biomedical Sciences, the University of Hong Kong, Hong Kong SAR, China; 11 Department of Computer Science, the University of Hong Kong, Hong Kong SAR, China; 12 Department of Respiratory Medicine, Wuhan Children's Hospital, Tongji Medical College, Huazhong University of Science and Technology, Wuhan, Hubei, China; 13 Department of Haematology, Wuhan Children's Hospital, Tongji Medical College, Huazhong University of Science and Technology, Wuhan, Hubei, China; 14 Department of Paediatrics, Affiliated Taihe Hospital of Hubei University of Medicine, Shiyan, Hubei, China; 15 The Pritzker school of Molecular Engineering, the Ben May department of Cancer Research, The University of Chicago, Chicago, USA; 16 Department of Pediatric, Third People's Hospital of Shenzhen, Second Affiliated Hospital of Southern University of Science and Technology, Shenzhen, Guangdong, China; 17 Department of Pathology, Fuyang People's Hospital, Anhui, China; 18 Australian Institute for Bioengineering and Nanotechnology (AIBN), The University of Queensland, Brisbane, Australia; 19 Department of Pathology, Intervention and Cell Therapy Center, Peking University Shenzhen Hospital, Shenzhen, China

**Keywords:** COVID-19, SARS-CoV-2 virus, children, lung development, vulnerability, *ACE2*, lung progenitor cells, single cell RNA sequencing, pluripotent stem cells

## Abstract

**Introduction:** An increasing number of children with severe coronavirus disease 2019 (COVID-19) is being reported, yet the spectrum of disease severity and expression patterns of angiotensin-converting enzyme 2 (ACE2) in children at different developmental stages are largely unknow.

**Methods:** We analysed clinical features in a cohort of 173 children with COVID-19 (0-15 yrs.-old) between January 22, 2020 and March 15, 2020. We systematically examined the expression and distribution of *ACE2* in different developmental stages of children by using a combination of children's lung biopsies, pluripotent stem cell-derived lung cells, RNA-sequencing profiles, and *ex vivo* SARS-CoV-2 pseudoviral infections.

**Results:** It revealed that infants (< 1yrs.-old), with a weaker potency of immune response, are more vulnerable to develop pneumonia whereas older children (> 1 yrs.-old) are more resistant to lung injury. The expression levels of *ACE2* however do not vary by age in children's lung. *ACE2* is notably expressed not only in Alveolar Type II (AT II) cells, but also in *SOX9* positive lung progenitor cells detected in both pluripotent stem cell derivatives and infants' lungs. The *ACE2^+^SOX9^+^* cells are readily infected by SARS-CoV-2 pseudovirus and the numbers of the double positive cells are significantly decreased in older children.

**Conclusions:** Infants (< 1 yrs.-old) with SARS-CoV-2 infection are more vulnerable to lung injuries. *ACE2* expression in multiple types of lung cells including *SOX9* positive progenitor cells, in cooperation with an unestablished immune system, could be risk factors contributing to vulnerability of infants with COVID-19. There is a need to continue monitoring lung development in young children who have recovered from SARS-CoV-2 infection.

## Introduction

The rapid spread of coronavirus disease 2019 (COVID-19) has resulted in a global health crisis, the like of which hasn't been seen in over a century. By 29^th^ October, the number of confirmed cases had surpassed 45 million globally with more than 1,180,000 deaths (https://coronavirus.jhu.edu) 1. Compared with the adult population, children with COVID-19 generally display milder symptoms and lower mortality 2, 3. Our recent report indicates a high frequency of oral-rectal transmission and generally mild clinical features among infected children 4. Nevertheless, with the rapid spread of COVID-19 globally, an increasing number of severe or even fatal infections is being reported in children 5-7. Although the mortality of COVID-19 in children is low, the health of children is concerned widely because COVID-19 is a novel disease, lacking evidence-base for formulating clinical decisions 8. Moreover, there is very little information about the underlying risk factors and spectrum of vulnerability to COVID-19 in children. The lung is the primary target of COVID-19 and ACE2 is known to be the cell-entry receptor of severe acute respiratory syndrome coronavirus 2 (SARS-CoV-2) 9-11. However, the risk factors for the COVID‐19 in children remain elusive. One possibility is that the milder disease in paediatric patients with SARS-CoV-2 infection might be associated with the different patterns of *ACE2* expression and immune responses 12. Recent reports indicate that the expression of *ACE2* in the lungs increases with age and is particularly high in adults who smoke 13. We sought to understand the clinical characteristics of paediatric patients with COVID-19 and whether expression of *ACE2* in lungs was associated with the disease prognosis in children. We systematically analyzed the clinical features of 173 children with COVID-19 including laboratory parameters of the immune system. The respiratory system is the most vulnerable target of SARS-CoV-2. Lung development begins at an early embryonic stage and continues after birth until adolescence 14-16. Lung progenitor cells are essentially involved in lung branching morphogenesis, cell growth, maturation, injury, and repair 17. Therefore, we also examined the profiles of *ACE2* expression in lung progenitor cells at embryonic-like stage by using hESC-derived lung cells, at infants and older children stages by using lung biopsy tissues respectively. Finally, we employed SARS-CoV-2 pseudoviral infection in living lung tissues, to study the correlation of *ACE2* expression and SARS-CoV-2 infection in children at different ages.

## Methods

### Study Design and Patients

Paediatric patients with COVID-19 were included from four hospitals in China, including the Third People's Hospital of Shenzhen (Shenzhen, Guangdong), Taihe Hospital of Hubei University of Medicine (Shiyan, Hubei), Wuhan Children's Hospital (Wuhan, Hubei), and Guangzhou Women and Children's Medical Center (Guangzhou, Guangdong). Patients who fulfilled the following criteria were included: (1) diagnosed with laboratory-confirmed COVID-19 according to the WHO guideline 18 and the recommendation of the National Health Commission of the People's Republic of China (NHC) 19. Patients with COVID-19 confirmed by two positive qPCR results but without pneumonia or respiratory symptoms were considered as asymptomatic cases. Those with upper respiratory signs only but no pneumonia evidence on CT image were classified as upper respiratory tract infection (URTI). Patients with pneumonia evidence on CT image with or without fever and respiratory symptoms were deemed to have pneumonia. Severe infection was diagnosed in individuals who fulfilled one of the following criteria: (1) respiratory distress with respiratory rate ≥ 30 times/min (aged > 5 years), ≥ 40 times/min (aged 1-5 years), ≥ 50 times/min (aged 2-12 months), or ≥ 60 times/min (aged < 2 months); (2) fingertip oxygen saturation ≤ 93% at rest; (3) partial pressure arterial oxygen: fraction of inspired oxygen ratio (PaO_2_/FiO_2_) ≤ 300 mm Hg (1 mm Hg = 0.133 kPa); or (4) obvious progression > 50% of lesions over 24-48 hrs on pulmonary imaging. Patients were considered to have critical infection if they had one of the followings: (1) respiratory failure and need for invasive mechanical ventilation; (2) shock; or (3) combined failure of other organs that required intensive care unit monitoring. Nasopharyngeal swabs or sputum were collected and SARS-CoV-2 was detected by real-time polymerase chain reaction. Infection was defined as at least two positive test results. Children required two negative test results prior to hospital discharge. No prematurely born children were included in this cohort study. This study was approved by the respective Institutional Review Board. Written informed consent was obtained from patients and/or guardians before data collected.

### Pseudovirus generation and viral infection

We first constructed full-length Spike protein gene of SARS-CoV-2 (QHR63250) in the pVAX-1 vector. The plasmid was confirmed by Sanger-sequencing analysis. The pseudovirus was generated by co-transfecting 293T cells with human immunodeficiency virus type 1 pNL4-3GFP**^+^**Env**^-^**Vpr**^-^** backbone and full-length S gene of SARS-CoV-2 or SARS-CoV expression plasmids as previously described 20. To titrate the virus, 96-well plates were seeded with 10,000 HEK293T-ACE2 cells per well in 10% FBS media. Cells were infected with serial dilutions of SARS-CoV-2 pseudovirus in a final volume of 200 μL. GFP^+^ Cells were evaluated using FACs analysis. Viral titre was calculated using the following formula: {(F × Cn) /V} × DF. 1x10^5^ TU virus was used for infection of tissue in a 24 well plate. Viral supernatant was collected 48 hours post-transfection and frozen at 150 °C. The pediatric lung tissues were cut into small tissue blocks (3-4 mm^2^) in RPMI medium and seeded in 24 well tissue culture plates and incubated for 48 hours with 500 μL of each pseudovirus supernatant. Supernatants without pseudovirus were used as negative control. The expression of green fluorescent protein (GFP), luciferase (LUC), and *ACE2* were examined using IHF staining of frozen sections of these lung tissues. We also used a vesicular stomatitis ΔG-luciferase virus pseudo-typed with the SARS-CoV-2 Spike protein (SARS-CoV-2-entry virus) as described previously 21-23. Luciferase activity was used to detect robust infection of SARS-CoV-2 pseudovirus in PSC-derived lung cells in Matrigel-coated culture condition.

### Pluripotent stem cells and lung differentiation

Protocols for maintenance of hESCs and generation of lung cells were slightly modified from previous studies 24, 25. At day 25, a portion of cells from late stage lung progenitor cells was harvested for examination. The other cultures were allowed to continue to mature lung cells (day 25 to 55) in maturation media for induction of the major types of cells found in human lung epithelium including alveolar type 1, type 2 cells, club cells, airway basal epithelial cells, goblet cells, ciliated cells; and the scarce populations of pulmonary neuroendocrine cells (PNECs) and Tuft cells.

### Immunofluorescence staining

Histological examination of biopsy samples collected from normal lung tissues adjacent to lesions was performed on paraffin-embedded samples as previously described 26. Lung tissues infected by SARS-CoV-2 pseudovirus were fixed for cryosection. For immunostaining, tissue sections were incubated with primary antibodies at 4 °C overnight and secondary antibodies at room temperature for 1 hour. Primary antibodies and secondary antibodies are described in the supplementary materials. Nuclei were counterstained by DAPI. To verify the function of the antibody, the immunostaining of ACE2 in testis and isotype IgG in lung was served as the positive and negative control respectively 27. *ACE2* positive cells in the lungs were randomly counted from different slide views on confocal microscopy. 20 views in each lung section were randomly captured and averaged cell numbers per 0.025 mm^2^ were used to define the distributions of *ACE2* positive cells in the lung tissues as described 28. At least nine patients' samples were used in each age group.

### Single cell analysis for pluripotent stem cell-derived lung cells

Single-cell capture, reverse transcription, cell lysis, and library preparation was performed using the Single Cell 3′ version 3 kit and chip according to the manufacturer's protocol (10x Genomics, USA). Single-cell suspensions were generated by dissociating the RUES2 (human embryonic stem cell line, hESC)-derived lung cells with 0.05% Trypsin/0.02% EDTA for 10-15 min, followed with passing through 40 µM strainer. The single cell suspension was achieved through sorting the dissociated cells in flow cytometry singlets. Cell count was adjusted to 1000-2000 cells per μL to target an estimated capture of 8000 cells. We filtered cells with less than 200 or more than 4000 genes detected as well as cells with mitochondria gene content greater than 20%, remaining 10238 and 11058 cells in 2 biological replicates, respectively. We normalized the UMI counts per cell by the size factors and took a logarithm transform using the *Normalize* function. We identified highly variable genes and selected the top 2000 variable genes for downstream analysis. We scaled the normalized counts and performed PCA on the highly variable genes using the *ScaleData* and *RunPCA* functions in the R Seurat package. We selected the top 40 PCs for downstream visualization and clustering analysis. We ran UMAP dimensional reduction using the *RunUMAP* function and then grouping cells of similar transcriptome profiles using the *FindNeighbors* function and *FindClusters* function (resolution = 0.15) in the R Seurat package. We identified marker genes using the *FindMarkers* function in the R Seurat package for further analysis. We re-identified marker genes for the merged 8 clusters and selected top TF and surface marker genes per cluster for heatmap plot using the *DoHeatmap* function in the R Seurat package. The rest plots were generated using the R ggplot2 package. scRNA-seq data is uploaded to the GEO repository database with accession number GSE162936.

### Quantification and Statistical Analysis

**Figure 1:**
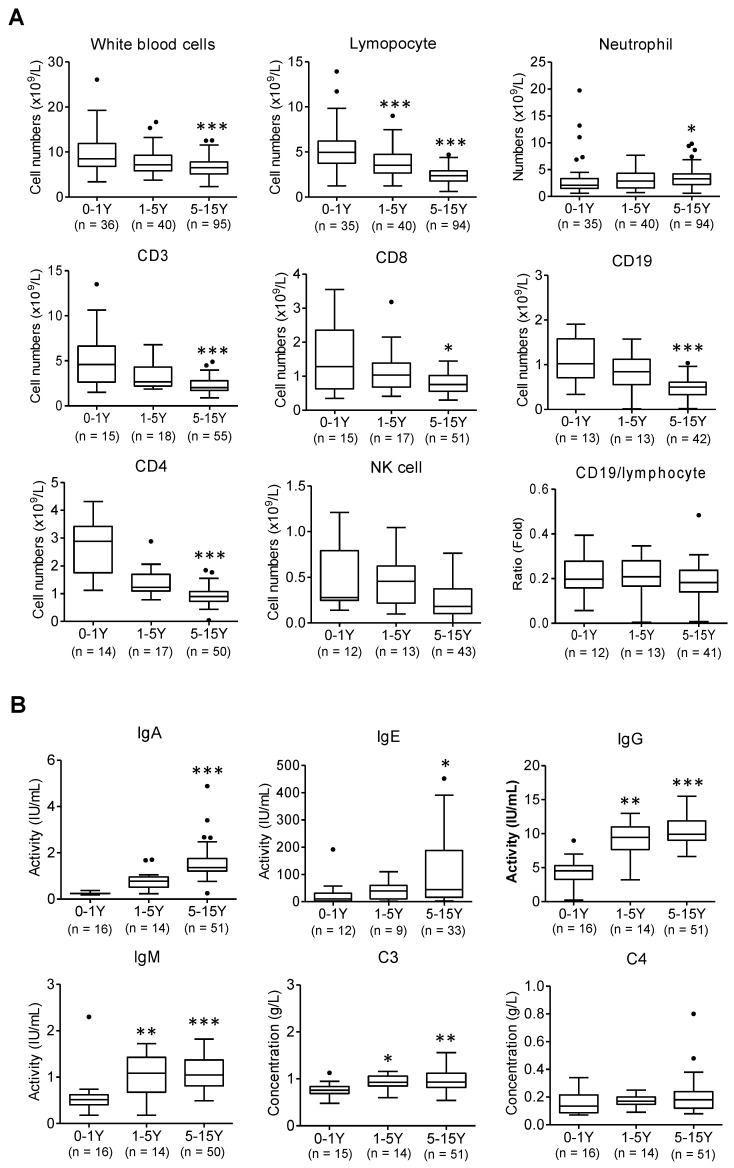
** Abnormal laboratory parameters in infected children.** (A) The peripheral blood samples and lymphocyte subpopulation were compared in infants (below 1 yrs.-old), younger children (1-5 yrs.-old), and older children (5-15 yrs.-old). Kruskal-Wallis test was used to examine the tendency of changes with increased ages. The total white blood cells, lymphocytes, CD3, CD8, CD19, CD4, NK cells were significantly decreased (all *p* < 0.05) but neutrophil was increased (*p* < 0.05); CD19/lymphocytes was not changed (*p* > 0.1); following Dunns test was used for pairwise comparison, *, *p* < 0.05, ***, *p* < 0.001 *vs*. infants (0-1 yr.). (B) The levels of immunoglobulin A (Ig A), Ig E, Ig M, Ig G and complement3 (C3), C4 were compared in infants (below 1 yrs.-old), younger children (1-5 yrs.-old), and older children (5-15 yrs.-old). Kruskal-Wallis test was used to examine the tendency of changes with increased ages. The levels of IgG, E, A, M and C3 were significantly increased (all *p* < 0.05) except C4 not changed (*p* > 0.1); following Dunns test was used for pairwise comparison *, *p* < 0.05, **, *p* < 0.01, ***, *p* < 0.001 *vs.* infants (0-1yr)

**Figure 2:**
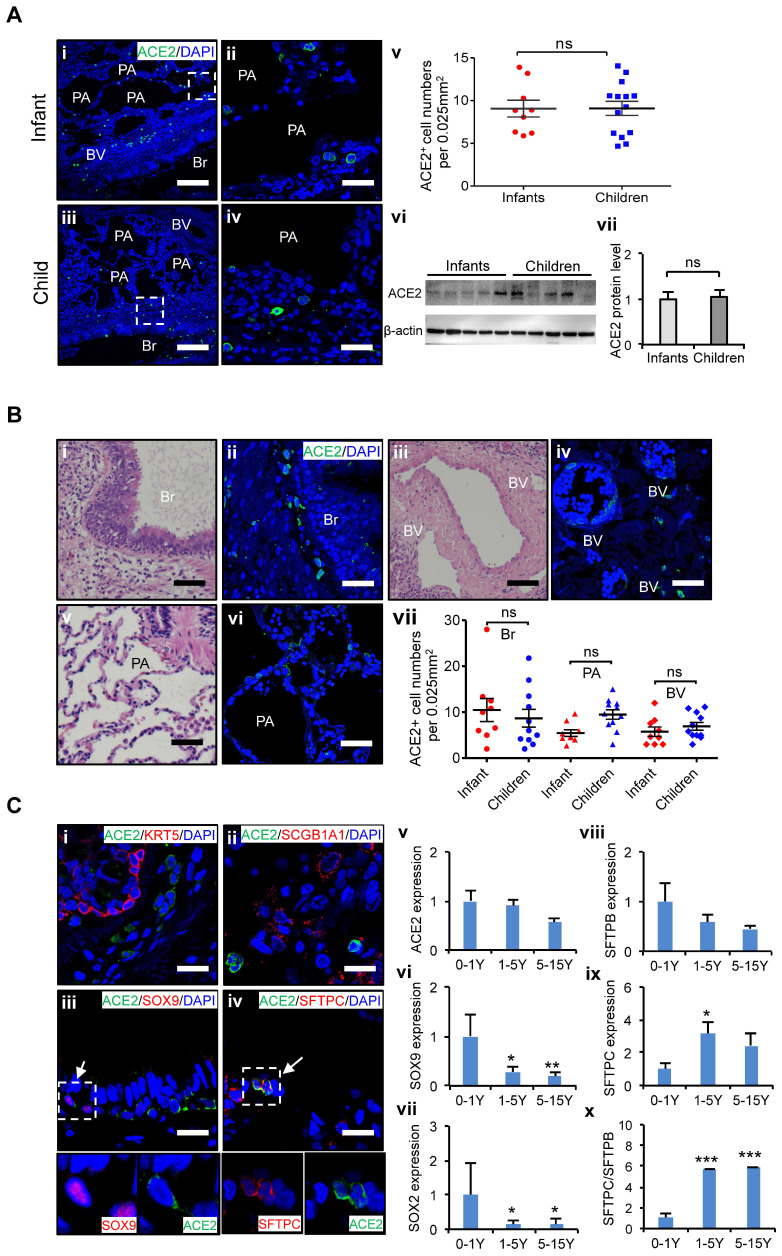
** Characteristics of ACE2 and lung progenitor cells distributed at different stages of children's lungs.** (A) i-iv. Representative photos showing expression and distribution of *ACE2* positive cells in lung tissues of infant and children. Scale bars = 50 µm. v. Comparison of* ACE2* positive cell numbers in lungs generally between infants (under 1 yrs.-old, n = 9) and older children (1-15 yrs.-old, n = 14), unpaired *t* test, *p > 0.05*. vi. ACE2 protein levels by western blotting between infants (under 1 yrs.-old, n = 5) and older children (1-15 yrs.-old, n = 5). vii. Statistical results of western blot, n = 5, unpaired *t* test, *p > 0.05*. (B) Distribution of *ACE2* positive cells in the different regions of lung. i, iii, v. HE staining showed the typical structures of children's lungs, including bronchus (Br), pulmonary alveolus (PA), and blood vessels (BV). Scale bar = 100 µm. ii, iv, vi. The immunostaining against *ACE2* positive cells at the regions of Br, PA, and BV. Scale bar = 50 µm. vii. Statistical analysis of *ACE2* positive cells distributed at Br, PA, and BV. N = 20, One-way ANOVA following Turkey's test, *p* > 0.05. (C) Expression of *ACE2* and lung progenitor markers. i-iv. The immunostaining showed the co-localization of ACE2 with SOX9 and SFTPC respectively at the bronchial-alveolar junction in lungs of children. ACE2 was rarely detected in KRT5 and SCGB1A1 expressed cells. Scale bar = 25 µm. v. The trend changes of *ACE2* expression in children's lungs with different ages measured by qPCR; vi-vii. Expression of lung progenitor genes *SOX9* and *SOX2* in different age group by qPCR. viii-x. Expression of *SFTPB*, *SFTPC* and ratio of *SFTPB*/*SFPTC* in different age group by qPCR. 0-1 yrs.-old group, n = 6; 1-5 yrs.-old group, n = 7; 5-15 yrs.-old group, n = 5; One-way ANOVA was used to examine the tendency of changes with increased ages, The levels of SOX9, SOX2, SFTPB, SFTPC, and SFTPB/SFTPC were significantly changed (all *p* < 0.05) except ACE2 not changed (*p* > 0.05); following Newman-Keuls test was used for pairwise comparison, *, *p* < 0.05, **, *p* < 0.01, ***, *p* < 0.001, *vs.* infants (0-1yr).

Age distribution and clinical severity was tested by Chi-square test or Fishers exact test in Table 1. For the analysis of blood cells and immunoglobulins in Figure 1, Kruskal-Wallis test was used. The expressions of ACE2^+^ cell in Figure 2A were analysed by unpaired *t* test. The distribution of ACE2^+^ cell in Figure 2B were compared by One-way ANOVA. Other statistical analyses were performed using One-way ANOVA or two-tailed *t* test. Total 18 samples were examined by qPCR, One-way ANOVA following Newman-Keuls test was performed. Data are presented as mean ± SD or SEM where indicated. *p* < 0.05 was considered significant. The SPSS software (version 20.0, IBM, Armonk, NY, USA) was used for statistical analysis. No outliers were excluded from these analyses.

## Results

### Characteristics of patients

In this retrospective study, 173 paediatric patients (91 males and 82 females) with COVID-19 were included from January 22 to March 15, 2020 (Figure S1). The patients were grouped into infants (0-1 yrs.-old), younger children (1-5 yrs.-old), and older children (5-15 yrs.-old) by ages (Table 1) 29.

Among infected children, 35 asymptomatic patients (20.2%) were included, far more than the counterpart in adults 3. Pneumonia was diagnosed in 105 patients (60.7%), but no death case was reported in our cohort. This is consistent with previous reports that the mortality of infected children was significantly lower than older adults 3. In all infected children, 20.2% children were asymptomatic, 19.1% children were presented with upper respiratory tract infection (URTI) and 60.7% children were shown evidence of lower respiratory tract infection (pneumonia) (Table 1). It suggests that children are also susceptible to SARS-CoV-2 infection although the mortality is low. There was no remarkable difference in genders on the distribution of age (Chi-square test, *p* = 0.272) (Table 1). In all patients, of note, asymptomatic patients (27/35; 77.1%) and URTI patients (24/33; 72.7%) were distributed mainly in older children groups (5-15 yrs.). In infected patients, 88.8% (32/36,) infants (0-1 yrs.) were developed into pneumonia whereas 68.3% (28/41) younger children (1-5 yrs.) and 46.9% (45/96) older children (1-5 yrs.) were presented with pneumonia (Chi-square test,* p* = 0.001) (Table 1).

The cell number of total white blood cells, T lymphocytes (CD3, CD4, and CD8), B lymphocytes (CD19), and Natural killer (NK) cells was gradually reduced with increased ages from infant patients (0-1 yrs.) to older children (1-5 yrs. and 5-15 yrs.) except neutrophils (Figure 1A, *, *p* < 0.05, ***, *p* < 0.001 *vs.* infant group). Meanwhile infant patients displayed a weaker potency of immune response than that of older children, as evidenced by a substantially weaker response of immunoglobin (Ig) A, Ig E, Ig M, Ig G and complement 3 (C3) (Figure 1B, *, *p* < 0.05, **, *p* < 0.01, ***, *p* < 0.001 *vs.* infant group). The viral infection provokes host immune responses reflected by reduced immune lymphocytes and increased immune responsive factors in blood.

However, compared to older children, it reveals lower levels of immune responsive factors following SARS-CoV-2 infection observed in infants (Figure 1B). Infant's (0-1 yrs.) immunity largely relies on the maternal resources before their own immune system is fully developed. These results also indicate that infants with immunocompromised state may increase risk of severe illness from COVID -19.

Overall, systematic analysis of 173 children with COVID-19 indicated that infants were more likely to develop pneumonia. Mild and asymptomatic patients were more common in older children, suggesting a higher risk of disease transmission. In addition, laboratory findings indicated that the immune responses in infant patients (0-1yrs.) has not been fully established which contributed to a higher risk of developing pneumonia after SARS-CoV-2 infection.

### The expression of ACE2 in different developmental stages of children

The lung is a main target of SARS-CoV-2, and ACE2 is the cell-entry receptor of SARS-CoV-2 30. Human lung development begins at an early embryonic stage and continues in children after birth until adolescence, albeit at a slower rate after 1 yr. 14, 15. To identify cell types potentially attacked by SARS-CoV-2, we examined the *ACE2* expression and distribution in children's lungs at different developmental stages.

At first, we examined the distribution of *ACE2* positive (ACE2^+^) cells in the lungs of age-matched children's biopsies. To minimize interference from individual variants, at least nine biological replicates of lung samples were used in each age group. Twenty views in each lung sections were counted, and averaged cell numbers per 0.025 mm^2^ were used to define the distributions of ACE2^+^ cells in the lung tissues as described 28. *ACE2* expression in the lungs of infants, and children up to 15 years of age was evaluated. It revealed that the protein levels of ACE2 in the lung did not significantly vary by age in children's lungs (Figure 2A), indicating all children in different ages could be similarly susceptible of SARS-CoV-2 infection. Furthermore, our results showed that ACE2^+^ cells were widely distributed throughout the whole lung including the bronchus and alveolus regions (Figure 2B), suggesting that these two regions are the high-risk zones targeted by SARS-CoV-2 infection. The mRNA expression level of bronchus progenitor marker genes, *SOX9* and *SOX2*, sharply decreased (Figure 2C vi and vii); in contrast, the ratio of *SFTPC*/*SFTPB*, the hallmark of maturation of AT II cells in the lungs, gradually increased with age (Figure 2C viii-x, *p* < 0.05). Importantly, *ACE2* expression was detected in some of *SOX9* positive (SOX9^+^) lung progenitor cells in infants' lungs (Figure 2C iii) but not SOX2 positive cells (Data not shown). In addition, *ACE2* was also expressed in *SFTPC* positive (SFTPC^+^) cells (Figure 2C iv), the AT II cells, but not SFTPB positive cells (Data not shown). SFTPC^+^ ATII cells were reported as a progenitor cells with a potential to differentiate into AT I cells during lung injury-repair or disease 31-33. However, *ACE2* was rarely detected in *SCGB1A1*-expressed epithelial cells and *KRT5* positive airway basal progenitor cells. These results indicated that *ACE2* was specifically expressed in certain types of lung progenitor cells in infants, raising concerns that SARS-CoV-2 may also attack lung progenitor cells of them.

Taken together, ACE2*^+^* cells are widely distributed in children lungs, and the expression levels of *ACE2* do not vary remarkably by ages in children. However, *ACE2* is expressed not only in AT II cells but also in some SOX9^+^ lung progenitor cells in infants. A higher proportion of SOX9^+^ lung progenitor cells in infant stage raises concerns that SARS-CoV-2 may also attack lung progenitor cells in neonates and infants.

### The differential expression of *ACE2* in human pluripotent stem cell-derived lung cells

**Figure 3:**
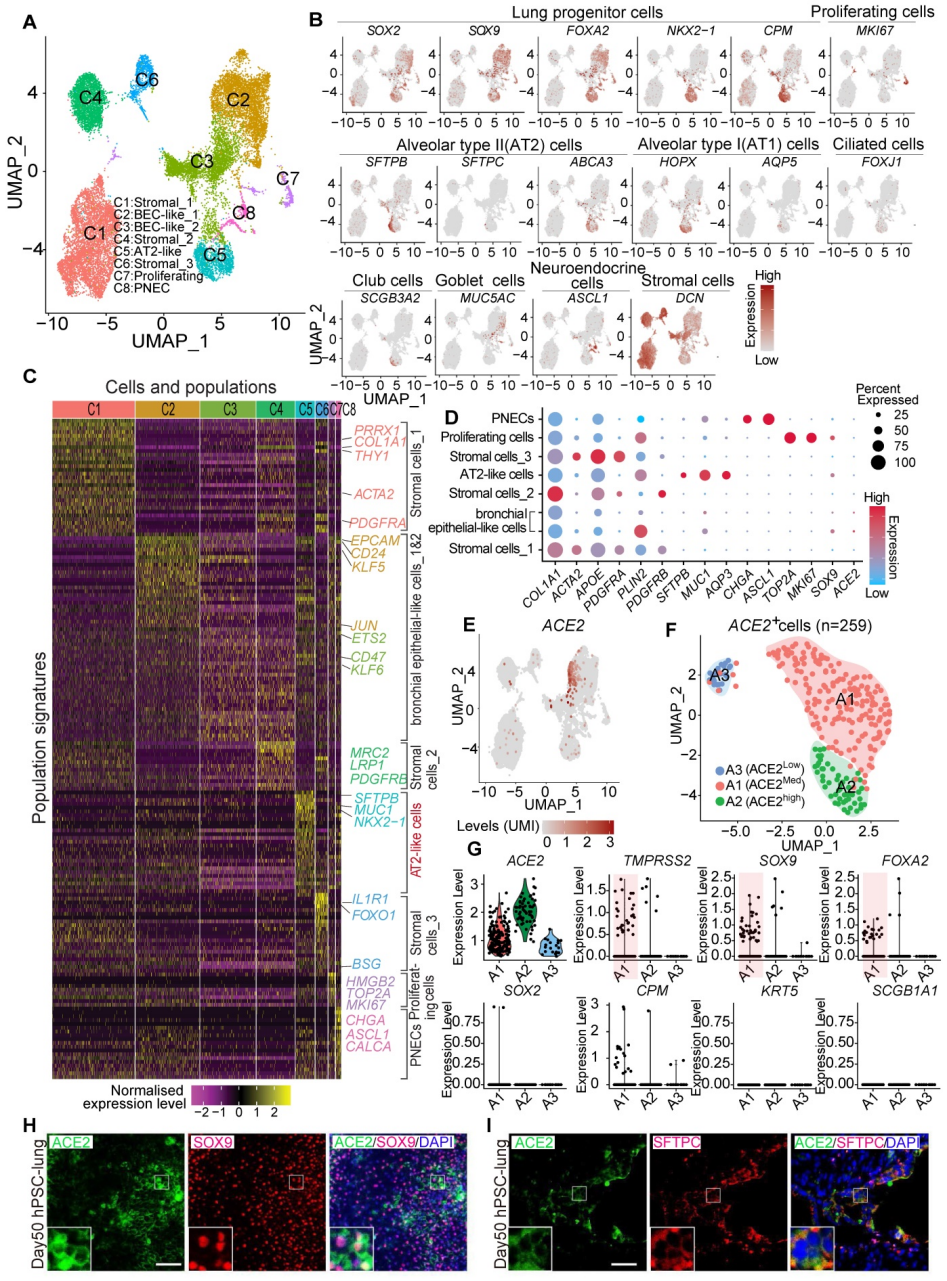
** ACE2 expression and distribution in hESC-derived lung cells.** (A) Single cell RNA sequencing of differentiated lung cells. UMAP of hESC-derived lung cells at day 50, colored and annotated with cluster 1-8. Cells were collected and pooled from n = 2 biological replicates. (B) Expression markers of lung cells in individual UMAPs. Relative expression of each gene ranges from low (grey) to high (dark red) as indicated. (C) Heatmap of top TF or surface marker genes in each cluster. Relative expression of each gene ranges from low (purple) to high (yellow) as indicated. (D) Dot plot shown the expression and percentage of signature genes in each cluster. Dot size indicates the percentage of indicated gene in each population. (E) UMAP of ACE2 expressed cells in hESC-derived lung at day 50. (F) ScRNA-seq data analysis of ACE2^+^ cells. UMAP of 259 ACE2^+^ cells (ACE2^+^, UMI count > 0), colored and annotated with cluster 0-2. Expression of selected genes in each cluster in UMAPs. Relative expression of each gene ranges from low (grey) to high (red) as indicated. (G). The violin plot shows the co-expression of ACE2 and other lung progenitor cells in each cluster. (H) Immunostaining of ACE2 and SOX9 in hESC-derived lung cells at day 50. Scale bar = 50 μm. (I) Immunostaining of ACE2 and SFTPC in hESC-derived lung cells at day 50. Scale bar = 50 μm.

Due to the difficulty of obtaining normal lung tissue from clinical samples, and to avoid individual variation of lung biopsies, we applied the lung lineage cells derived from human embryonic stem cells (hESCs) to model the expression pattern of SARS-CoV-2 receptors in lung cells at early developmental stages. hESCs were first differentiated into definitive endoderm, anterior foregut endoderm (AFE), then into AFE/lung progenitor cells and lung progenitor cells by day 15-25 (Figure S2A-B). The lung progenitor cells were further differentiated to the major types of cells found in human lung epithelium by Day 50-55 (Figure S2A) 24, 25, 34. We further characterized the hESC-derived lung cells at day 50 by single cell RNA (scRNA) sequencing. Two major distinct populations, stromal cells, and epithelial cells, were identified (Figure S3A-H) and 8 sub-clusters were annotated by their signature genes (Figure 3A and 3C). scRNA analysis showed that expression of *ACE2*, *SOX2*, *SOX9*, *SFTPB*, and *ABCA3* was distributed in the populations of lung epithelial cells (Figure 3B, Figure S3H). Main marker genes of stromal cells, such as *COL1A1*,* PDGFRA*,* APOE*, and *DCN* were mainly enriched in cluster 1, 4, and 6 (Figure 3B-D). AT II cell-related markers such as *SFTPB*, *SFTPC*, *MUC1*, and *ABCA3* were mainly expressed in cluster 5 (Figure 3B-D).

Further clustering of scRNA profiles by UMAP revealed that the expression of *ACE2* and *TMPRSS2* were mainly enriched in the clusters representing distal (SOX9^+^) lung progenitor cells and AT II cells, but not in SOX9^+^SOX2^+^, SOX2^+^, SFTPC^+^HOPX^+^, SOX9^+^HOPX^+^, or SFTPC^+^SOX9^+^ progenitor cells. (Figure 3D-G and Figure S3I-L). Immunostaining results further validated the expression of *ACE2* in SOX9^+^ cells and SFTPC^+^ cells (Figure 3H). In consistence with the results from *in vivo* studies (Figure 2), the *in vitro* lung differentiation model confirmed the expression of *ACE2* in lung SOX9^+^ progenitor and AT II cells, suggesting the high risk that SARS-CoV-2 can attack lung progenitor cells in infants.

### *Ex vivo* infection by SARS-CoV-2 pseudovirus in PSC-derived lung progenitor cells and pediatric lung tissues

**Figure 4:**
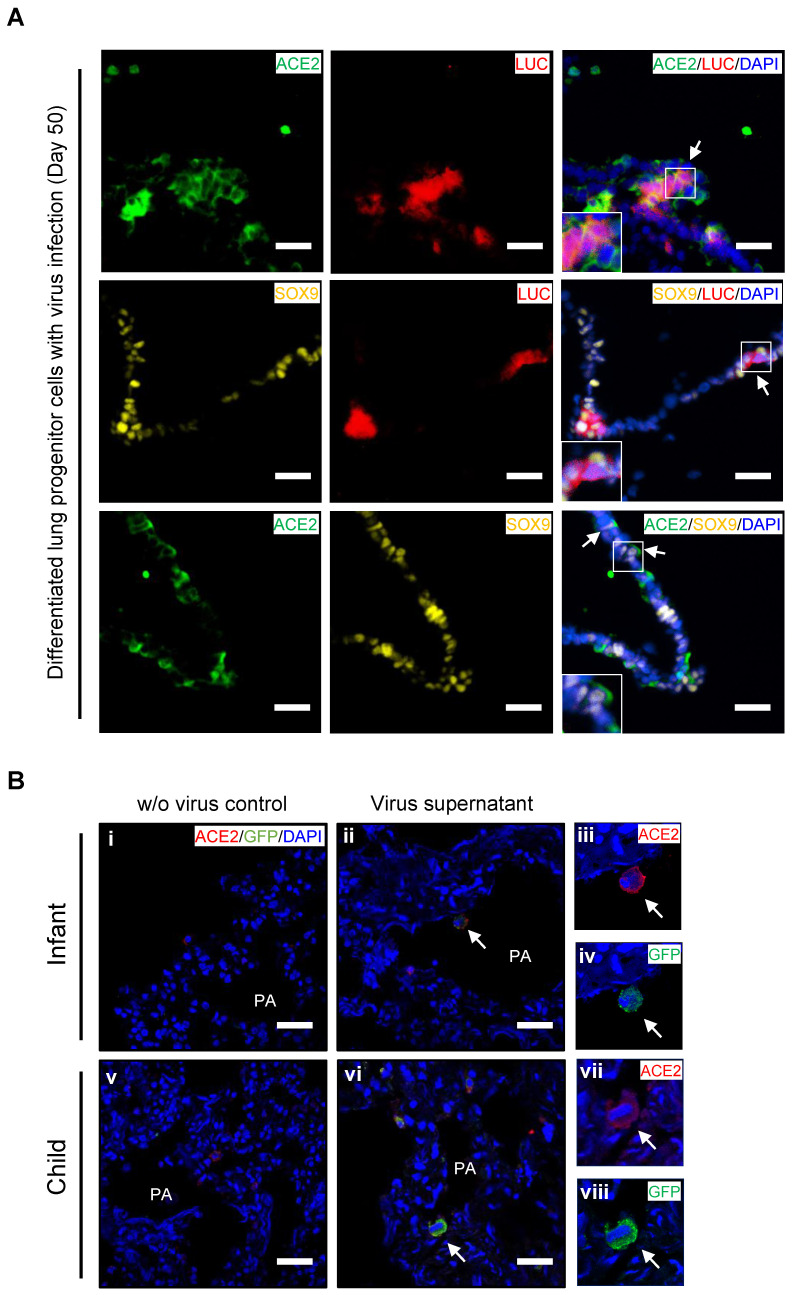
***Ex vivo* infection by SARS-CoV-2 pseudovirus in lung tissues and hESC-derived lung progenitor cells.** (A)* Ex vivo* infection of hESC-derived lung progenitor cells. Matrigel-coated lung progenitor cells were grown in a 3D condition for one week. The cells were infected with nCoV-LUC-pseudovirus then embedded and sectioned for immunostaining. The co-localizations of ACE2/LUC, SOX9/LUC, and ACE2/SOX9 were detected in nCoV-pseudovirus-infected lung progenitor cells. Scale bar = 25 μm. (B) *Ex vivo* infection of lung tissues from infant and older children. Lung tissue from an infant and an older child were exposed to supernatants without nCoV-pseudovirus as control (i-iii). Lung tissue (iv-vi) from an infant and an older child were infected by nCov-GFP-pseudovirus supernatant (iv-xii). Immunostaining against ACE2 (red) and GFP to detect nCOV in all lung biopsies. The co-localization of GFP and ACE2 (arrow heads) indicated infection with nCoV-pseudovirus (viii, x, and, xii) in lung cells with ACE2 expression (vii, ix, and, xi). Scale bar = 50 µm.

To confirm whether lung progenitor cells could be infected by SARS-CoV-2, we first used a vesicular stomatitis ΔG-luciferase virus pseudo-typed with the SARS-CoV-2 Spike protein (SARS-CoV-2-entry virus). Robust luciferase activity was readily detected in the human ESC-derived lung cells infected with SARS-CoV-2-entry virus. The co-localization of ACE2, LUC (Luciferase), and SOX9 indicated the successful infection of SARS-CoV-2 in ACE2^+^/SOX9^+^ progenitor of lung (Figure 4A). To determine whether native infant or children lung cells that express *ACE2* are the target of SARS-CoV-2 virus, we next infected *ex vivo* live lung tissues with SARS-CoV-2-GFP pseudovirus (nCov-GFP-pseudovirus). Lung tissues were obtained from biopsies of infants or children who were undergoing surgeries for lung abscess. After 48 hours of nCov-GFP-pseudovirus infection at a concentration of 1×10^5^ TU, lung tissues were washed, fixed, and cryo-sectioned for immunostaining. ACE2 was detected in lung tissues (Figure 4B i and v). These lung tissues were used for control (Figure 4B i and v) or for nCov- GFP-pseudovirus infection. After lung tissues were exposed to nCov-GFP-pseudovirus, it reveals that ACE2 expressed cells (red) were infected by SARS-CoV-2 pseudovirus (green) as evidenced by the co-location of ACE2 and nCov (Figure 4A ii-iv and vi-viii). This suggests that infants and children, like adults, are susceptible to SARS-CoV-2 infection. It also indicates that in children, infection causes milder lung damage and lower mortality, possibly due to their immune response to SARS-CoV-2 invasion and ability of progenitor cells to rapidly regenerate and repair, rather than their resistance to cell-entry by the virus.

## Discussion

With the ongoing pandemic of COVID-19 globally, increasing numbers of infected and critically ill children are being reported although their mortality remains lower than that of adults. Few data are available about the spectrum of disease severity in children, and its correlation with the expression profiles of *ACE2* in lungs at different developmental stages. This study had several key findings.

First, infants were particularly vulnerable to severe lung injury and more likely to develop a pneumonia among children with COVID-19, while asymptomatic and URTI patients were common in children older than 1 yr. (Table 1). Infants present weaker immune responses to SARS-CoV-2 infection (Figure 1). This suggests that the immune system of infants is not well-established and insufficient to protect against SARS-CoV-2 infection, which may in part explain the increased risk of severe consequences of infants with SARS-CoV-2 infection. Notably, 77.1% (27/35) of asymptomatic patients were in children above 5 years of age, indicating these children may be in risk for SARS-CoV-2 transmission due to their actively interaction with their family and people at school.

Second, recent reports indicated *ACE2* was highly expressed in the sinonasal cavity and pulmonary alveoli, proposing a hypothesis that *ACE2* expression and distribution contribute to disease severities in paediatric patients with COVID-19 35. We firstly examined the distribution and expression of *ACE2* using age-matched children's lung tissues from neonates to 15 yrs.-old children systematically (Figure 2A-B). No remarkable difference of *ACE2* expression was found in lung tissues between infants and older children in mRNA and protein levels. The ACE2^+^ cells were similarly distributed in bronchus and alveolus between infants and older children as well but infants are more vulnerable to develop into severe condition. These findings indicate it is insufficient to prognose disease severity simply by the profiles of *ACE2* expression and distribution in patients with COVID-19.

Recent studies indicated that *ACE2* is also expressed in some pulmonary tract progenitor cells, i.e. airway secretory transitional cells 36. Since lung development continues after birth, particularly during the first year of life, it is essential to maintain sufficient lung progenitor cells for rapid lung development and repair 14, 15, 37. We therefore examined if the *ACE2* expression in lung progenitor cells may contribute to the different severity of disease between infants and older children with COVID-19.

We assessed the co-expression of lung progenitor genes and *ACE2* in age-matched lung tissues. SOX9^+^ lung progenitor cells were highly enriched in foetal stage during lung morphogenesis and branching, and lasted in neonate and infant stage, then disappeared quickly, which was further evidenced by qPCR results (Figure 2C vi) 38, 39. ACE2-expressed SOX9^+^ lung progenitor cells were presented in neonates and infants, but rare in older children, (Figure 2C i-iv). Reactivation of *SOX9* is also relative to regulate injury repair in alveolar homeostasis and regeneration 40, 41. To further verify the *ACE2* expression in SOX9^+^ progenitor cells and other lung cells in developing lungs, we used human pluripotent stem cell-derived lung cells as research resources. We differentiated hESC into lung lineage cells and harvested most types of lung cells (Figure 3, Figure S2, and Figure S3). scRNA-seq reveals *ACE2*/*TRMPRSS2* is abundantly expressed in SOX9^+^ lung progenitor cells (Figure 3H). Immunostaining results further confirm that *ACE2* is expressed in SOX9^+^ cells and AT II (SFTPC^+^) cells. SARS-CoV-2 pseudovirus infection was evident in all lung tissues and hESC-derived cells expressing *ACE2* (Figure 4). Of note, the first years of life are a critical period for lung development and *SOX9* plays a critical role for continuing development of lung. Compared with older children, the infant lung is underdeveloped, and a significant amount of lung progenitor cells are maintained for normal lung development. Although the long-term consequences of SARS-CoV-2 infection are unknown, it is likely that these ACE2^+^ lung cells including SOX9^+^ progenitor cells, will be attacked and damaged in the subsequent inflammatory response of SARS-CoV-2 infections 42.

There are several limitations in this study. First, the tissue samples for histological study were collected from normal tissues adjacent to lesions of lung abscess. Whether the *ACE2* expression is influenced under this condition needs further investigations. Second, our study demonstrated* ACE2* is expressed and distributed in pluripotent stem cells-derived lung cells. It is a first step toward understanding impacts of SARS-CoV-2 infection in *ex vivo* model of lung development, but is clearly simplified compared to fully functioning human lung systems in human. Third, bias exists between the investigation in SARS-CoV-2 pseudoviral infection models and real clinical case. In addition, besides of the pulmonary progenitor cells with *ACE2* expression, other progenitor or stem cells were rich in infant lungs. It is unclear in current study if other progenitor or stem cells will play a role to repair the injury induced by SARS-CoV-2 infection.

In summary, *ACE2* expression levels appear not vary remarkably by age in children. Infants with COVID-19 (< 1yrs.-old) were more vulnerable to severe lung injury. The expression of *ACE2* in multiple types of lung cells including SOX9^+^ progenitor cells and an unestablished immune system could be risk factors contributing to vulnerability of infants in SARS-CoV-2 infection. It is vital to perform a long-term monitoring of lung development in young children who have recovered from COVID-19.

## Supplementary Material

Supplementary figures, materials and methods.

## Figures and Tables

**Table 1:** Demographics and baseline characteristics of patients with COVID-19

	All patients (*n* = 173)	0-1 yrs. (*n* = 36)	1-5 yrs. (*n* = 41)	5-15 yrs. (*n* = 96)	*P* value
Characteristics					
Gender, *n* (%)					0.272
Males	91 (52.6)	15 (41.7)	21 (51.2)	55 (57.3)	
Females	82 (47.4)	21 (58.3)	20 (48.8)	41 (42.7)	
Disease severity, *n* (%)					0.001
Asymptomatic	35 (20.2)	2 (5.6)	6 (14.6)	27 (28.1)	
URTI	33 (19.1)	2 (5.6)	7 (17.1)	24 (25.0)	
Pneumonia	105 (60.7)	32 (88.8)	28 (68.3)	45 (46.9)	
Signs and symptoms, *n* (%)					
Fever	57 (32.9)	18 (50.0)	19 (46.3)	20 (20.8)	0.028
Cough	57 (32.9)	13 (36.1)	17 (41.5)	27 (28.1)	0.536
Diarrhea	7 (4.0)	5 (13.9)	0 (0.0)	2 (2.1)	0.006
Sore throat	6 (3.5)	0 (0.0)	1 (2.4)	5 (5.2)	0.338
ICU admission,* n* (%)	1 (0.6)	1 (2.8)	0 (0.0)	0 (0.0)	NA

Data were presented with patient's number (n) and percent (%). *P* values were calculated using Chi-square test or Fisher's exact test. URTI, upper respiratory tract infection; ICU, intensive care unit.
