# Supplementary material for: Clinical analysis and pluripotent stem cells-based model reveal possible impacts of ACE2 and lung progenitor cells on infants vulnerable to COVID-19
Source: Theranostics. 2021 Jan 1;11(5):2170–81. doi: 10.7150/thno.53136 (PMC7797681; doi:10.7150/thno.53136)
Supplement: Supplementary file 1 — Supplementary figures, materials and methods. [file thnov11p2170s1.pdf]

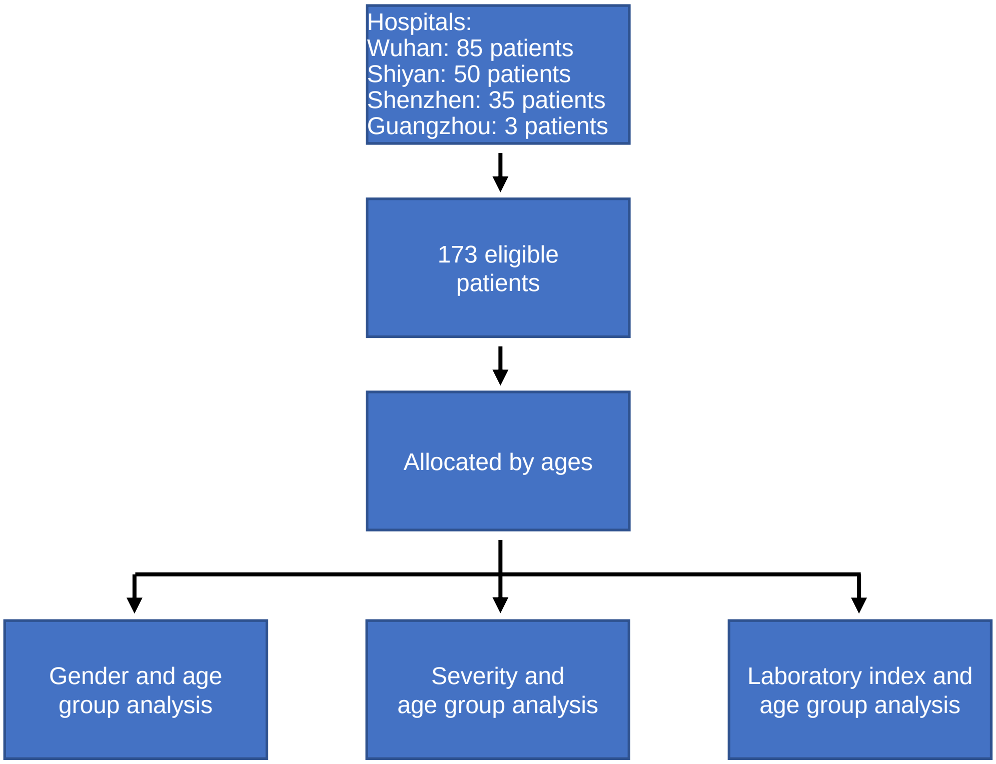
Hospitals:
Wuhan: 85 patients
Shiyan: 50 patients
Shenzhen: 35 patients
Guangzhou: 3 patients
173 eligible patients
Allocated by ages
Gender and age group analysis
Severity and age group analysis
Laboratory index and age group analysis

Figure S1. Diagram of patient enrolment and data analysis.

The flowchart presents the patients' recruitment and 173 patients were grouped into infants (0-1 yrs.-old), younger children (1-5 yrs.-old), and older children (5-15 yrs.-old) by ages to analyse the gender distribution, disease severity (Table 1), and laboratory findings (Figure 1).

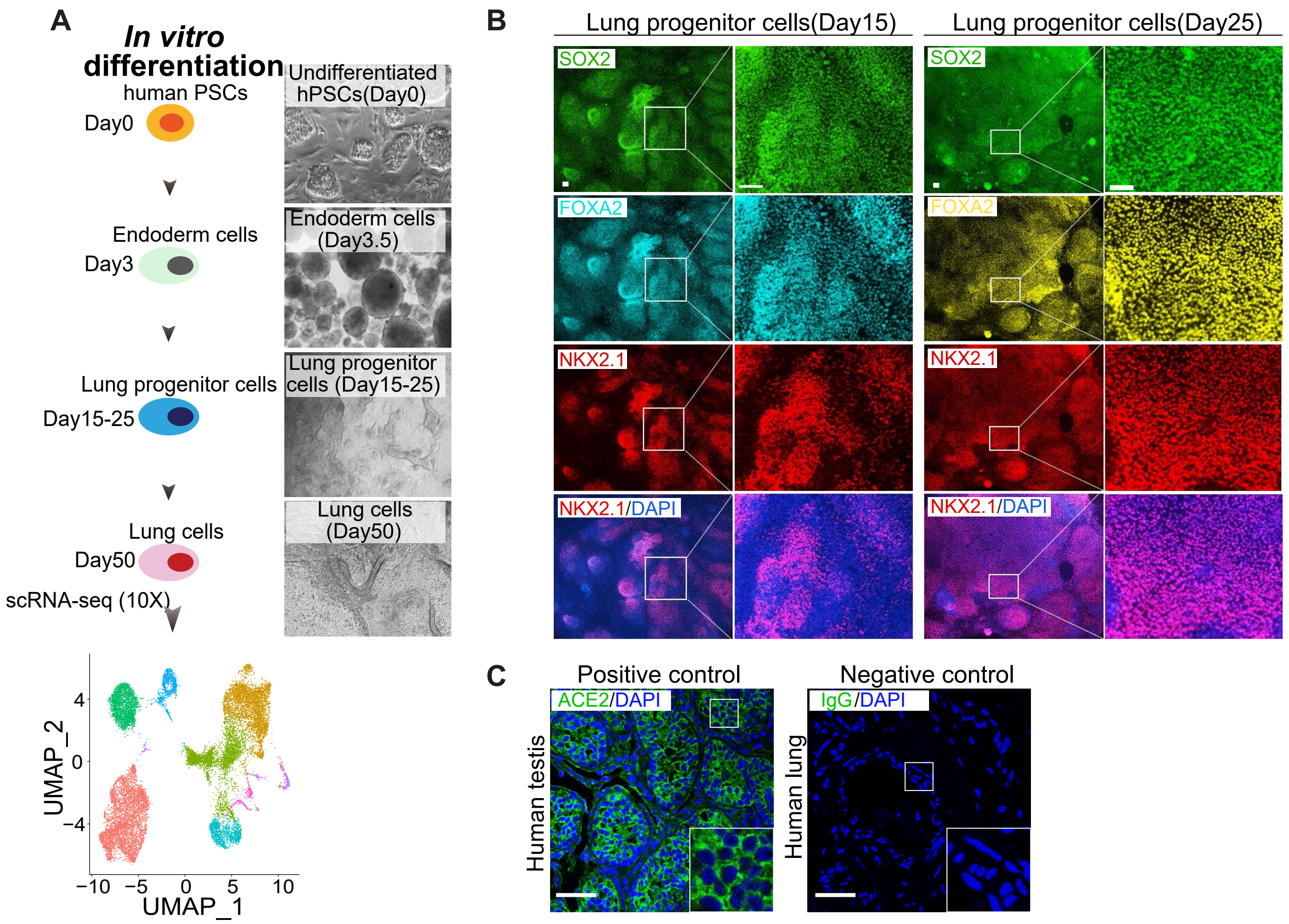
A
In vitro differentiation
human PSCs
Day0
Endoderm cells
Day3
Lung progenitor cells
Day15-25
Lung cells
Day50
scRNA-seq (10X)
Undifferentiated hPSCs(Day0)
Endoderm cells (Day3.5)
Lung progenitor cells (Day15-25)
Lung cells (Day50)
UMAP_2
UMAP_1
4
0
−4
−10
−5
0
5
10
B
Lung progenitor cells(Day15)
Lung progenitor cells(Day25)
SOX2
FOXA2
NKX2.1
NKX2.1/DAPI
C
Positive control
Negative control
ACE2/DAPI
IgG/DAPI
Human testis
Human lung

Figure S2. Directed differentiation of hESC into lung cells

(A). Scheme of directed differentiation of hESC into lung cells. Biomarkers and typical morphology at different differentiating stages were indicated.

(B) Representative and IF images in the hESC-derived cell cultures at day 15-25. The cells that are triple-positive for SOX2, FOXA2 and NKX2.1 at anterior foregut endoderm/lung progenitor stage or lung progenitor stage. Scale bars = 100 μm.

(C) The positive and negative controls of immunostaining. ACE2 stained in human testis was used as positive control, and IgG isotype stained in human lung was used as negative control. Scale bar = 50 μm.

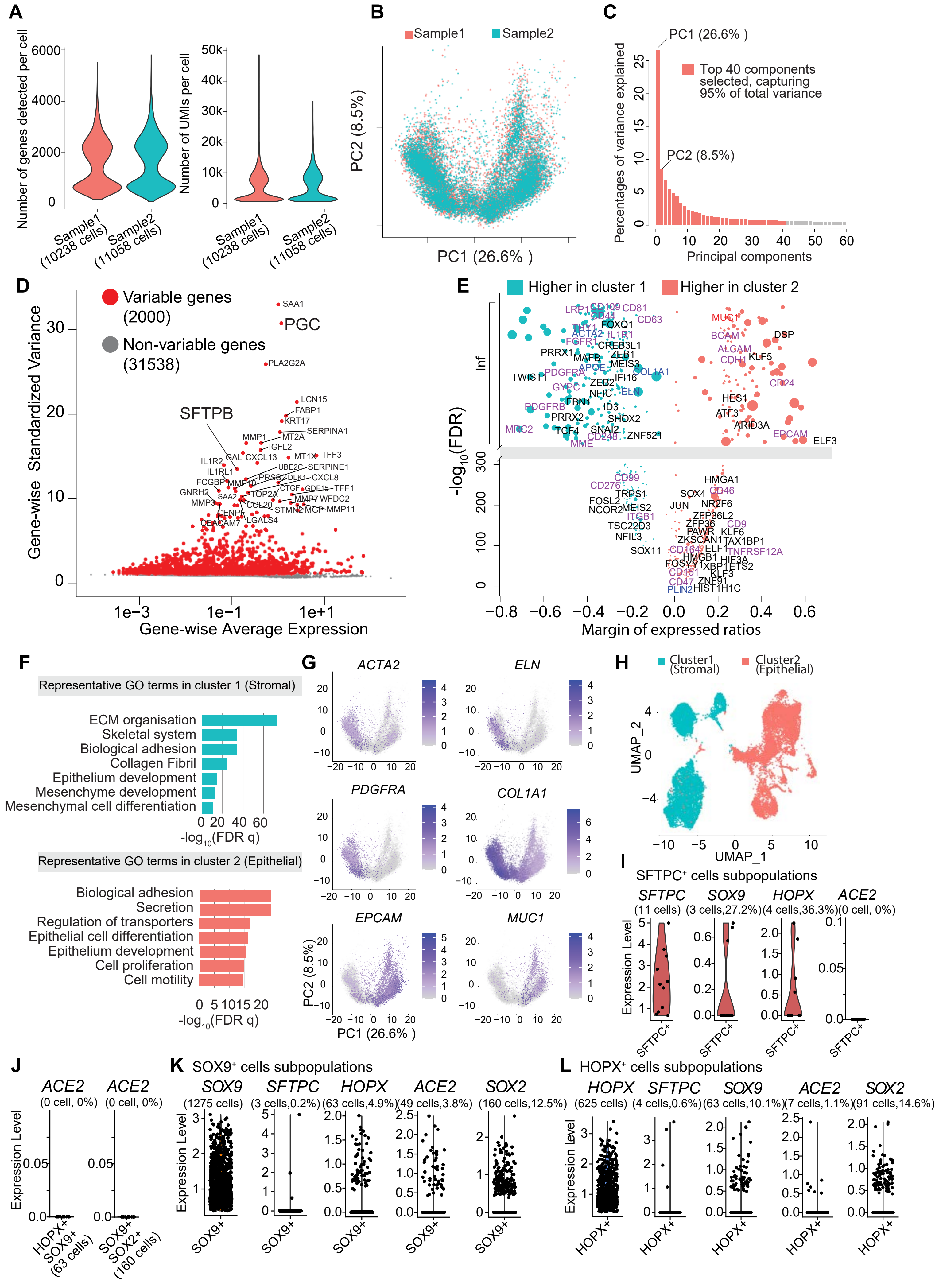
A
Number of genes detected per cell
Number of UMIs per cell
Sample1 (10238 cells)
Sample2 (11058 cells)
B
Sample1
Sample2
PC2 (8.5%)
PC1 (26.6%)
C
PC1 (26.6%)
PC2 (8.5%)
Top 40 components selected, capturing 95% of total variance
Percentages of variance explained
Principal components
D
Variable genes (2000)
Non-variable genes (31538)
SAA1
PGC
PLA2G2A
SFTPB
Gene-wise Standardized Variance
Gene-wise Average Expression
E
Higher in cluster 1
Higher in cluster 2
Margin of expressed ratios
F
Representative GO terms in cluster 1 (Stromal)
ECM organisation
Skeletal system
Biological adhesion
Collagen Fibril
Epithelium development
Mesenchyme development
Mesenchymal cell differentiation
Representative GO terms in cluster 2 (Epithelial)
Biological adhesion
Secretion
Regulation of transporters
Epithelial cell differentiation
Epithelium development
Cell proliferation
Cell motility
G
ACTA2
ELN
PDGFRA
COL1A1
EPCAM
MUC1
H
Cluster1 (Stromal)
Cluster2 (Epithelial)
UMAP_1
UMAP_2
I
SFTPC⁺ cells subpopulations
SFTPC (11 cells)
SOX9 (3 cells,27.2%)
HOPX (4 cells,36.3%)
ACE2 (0 cell, 0%)
Expression Level
J
ACE2 (0 cell, 0%)
ACE2 (0 cell, 0%)
HOPX+ SOX9+ (63 cells)
SOX9+ SOX2+ (160 cells)
K
SOX9⁺ cells subpopulations
SOX9 (1275 cells)
SFTPC (3 cells,0.2%)
HOPX (63 cells,4.9%)
ACE2 (49 cells,3.8%)
SOX2 (160 cells,12.5%)
L
HOPX⁺ cells subpopulations
HOPX (625 cells)
SFTPC (4 cells,0.6%)
SOX9 (63 cells,10.1%)
ACE2 (7 cells,1.1%)
SOX2 (91 cells,14.6%)

Figure S3. Single cell sequencing of hESC-derived cells

(A) Numbers of genes (left) and total UMIs (right) detected per biological replicate (n = 2).

(B) Principal component analyse shows that the two samples are representative to each other.

(C) Bar-chart showing the percentages of variance explained per each of the principal components, top 40 of which were selected for later dimension reduction approaches.

(D) Agnostic approach identified top 40 most informative genes (by dispersion) of the data.

(E) Volcano plot showing the most differentially expressed genes between cluster 1 and cluster 2, highlighting transcription factors (black) and surface markers (purple). Larger dot-size represents higher absolute value of $\log_2$ (fold-change). "Margin of expressed ratios is the difference between the percentage of cells expressing one gene in one cluster minus that in another cluster. Black texts are transcription factors. Red text genes are reported markers of lung epithelial cells, and blue text genes are reported markers of lung stroma cells.

(F) Representative gene-ontology (GO) terms enriched for the DEGs higher in cluster 1 (cyan) or in cluster 2 (orange).

(G) Representative markers selected from the DEGs between cluster 1 and cluster 2, and were also reported by Travaglini et al. 2019.

(H) Scatterplot showing the first two components a UMAP reduction result, wherein the colors refer to the two major clusters identified by PCA.

(I) The violin plot shows the expression of SOX9, HOPX, and ACE2 in $SFTPC^{+}$ subpopulation.

(J) The violin plot shows the expression of ACE2 in $HOPX^{+}SOX9^{+}$ and $SOX9^{+}SOX2^{+}$ cells.

(K) The violin plot shows the expression of SFTPC, HOPX, ACE2, and SOX2 in the $SOX9^{+}$ subpopulation.

(L) The violin plot shows the expression of SFTPC, SOX9, ACE2, and SOX2 in the $HOPX^{+}$ subpopulation.

Supplementary Methods and Materials

Key Resources Table

Antibodies

Goat polyclonal anti-ACE2, R&D system, Cat# AF933,

Rabbit monoclonal anti-KRT5, Abcam, Cat# ab52635,

Mouse monoclonal anti-SCGB1A1, Santa Cruz Biotechnology, Cat# sc-365992

Rabbit polyclonal anti-SFTPC, Abcam, Cat# ab90716,

Rabbit monoclonal anti-SOX9, Millipore, Cat# AB5535,

Rabbit polyclonal anti-GFP, Thermo Fisher Scientific, Cat# A-11122,

Mouse monoclonal anti-Luciferase, Thermo Fisher Scientific, Cat# 35-6700

Goat polyclonal anti-FOXA2, Santa Cruz Biotechnology, Cat# sc-6554

Rabbit polyclonal anti-SOX2, Abcam, Cat# ab97959,

Rabbit polyclonal anti-NKX2.1, Seven Hills Bioreagents, Cat# WRAB-1231

Kits

Trizol, Thermo Fisher Scientific, Cat# 15596026,

PrimeScript RT Reagent Kit with gDNA Eraser, Takara, Cat# RR047B,

Green Premix Ex Taq II (Tli RNase H Plus), Takara, Cat# RR820B,

QIAGEN Plasmid Maxi Kit (25), Qiagen, Cat# 12163,

H&E Staining Kit, Abcam, Cat# ab245880,

Cell Lines

HEK293T, ATCC, Cat# CRL-3216,

Oligonucleotides

SOX2: CCCATGCACCGCTACGACG

CGGACTTGACCACCGAACCC

SOX9: GAACGCCTTCATGGTGTGG

GGGTGGTCCTTCTTGTGCTG

SFTPB: CTTCCAGAACCAGACTGACTCA

GCTCGGAGAGATCCTGTGTG

SFTPC: GCAAAGAGGTCCTGATGGAG

TGTTTCTGGCTCATGTGGAG
ACE2: GGACCCAGGAAATGTTCAGA
GGCTGCAGAAAGTGACATGA

Method Details

RNA extraction and cDNA synthesis

Trizol reagent was used to extract total RNA of the lung samples according to the instructions. Briefly, 50-100 mg of lung tissues were homogenized in liquid nitrogen, then were transferred into 1.5 mL EP tubes filled with 1 mL trizol. The chloroform was added for vortex, which was followed with centrifuge (4°C, 12,000g, 15 mins). The supernatant was transferred into the new EP tubes, and the isopropanol was added to deposit the RNA. After the centrifuge and washing, total RNA was used for cDNA synthesis according to the instructions of reverse transcription kit of Takara.

qPCR

The qPCR was set and run according to the instruction of Takara Premix Ex Taq II. Briefly, 2 μL of cDNA was used as template to generate a 20 μL reaction system for each sample by mixing with the 0.4 μL primers (10 μM), 10 μL Takara SYSB Taq II mixture, and ddH2O. Step One & Step One Plus Real-Time PCR Systems (Thermo Fisher Scientific) was used for qPCR with the reaction condition: 95 °C 30 s, and 40 cycles of 95 °C 5 s, 60 °C 31 s. For data analysis, $2^{-\Delta\Delta CT}$ method was used.

H&E staining

The lung section was stained following the instruction of H&E staining kit. After the standard process of deparaffinization and hydration, the section was incubated with hematoxylin for 5 mins at room temperature, and followed with the incubation of bluing reagent (10-15 s). Then, the slide was rinsed by distilled water, and stained with eosin Y solution for 2-3 mins. Finally, the section could be mounted for observation after the standard rehydration.
